# Changing the role of pCR in breast cancer treatment - an unjustifiable interpretation of a good prognostic factor as a “factor for a good prognosis“

**DOI:** 10.3389/fonc.2023.1207948

**Published:** 2023-07-18

**Authors:** Nebojsa Ivanovic, Dragana Bjelica, Barbara Loboda, Masan Bogdanovski, Natasa Colakovic, Simona Petricevic, Milan Gojgic, Ognjen Zecic, Katarina Zecic, Darko Zdravkovic

**Affiliations:** ^1^ Department of Surgical Oncology, University Hospital Medical Center (UHMC) “Bezanijska kosa”, Belgrade, Serbia; ^2^ Department of Surgery, Faculty of Medicine, University of Belgrade, Belgrade, Serbia; ^3^ Faculty of Philosophy, Department of Philosophy, University of Belgrade, Belgrade, Serbia; ^4^ Clinic for Gynecology and Obstetrics, University Clinical Centre of Serbia, Belgrade, Serbia

**Keywords:** early breast cancer, pathologic complete response, neoadjuvant systemic therapy, adjuvant systemic therapy, prognostic factor, treatment guidelines

## Abstract

Pathologic complete response (pCR) after neoadjuvant systemic therapy (NAST) of early breast cancer (EBC) has been recognized as a good prognostic factor in the treatment of breast cancer because of its significant correlation with long-term disease outcome. Based on this correlation, pCR has been accepted by health authorities (FDA, EMA) as a surrogate endpoint in clinical trials for accelerated drug approval. Moreover, in recent years, we have observed a tendency to treat pCR in routine clinical practice as a primary therapeutic target rather than just one of the pieces of information obtained from clinical trials. These trends in routine clinical practice are the result of recommendations in treatment guidelines, such as the ESMO recommendation “…to deliver all planned (neoadjuvant) treatment without unnecessary breaks, i.e. without dividing it into preoperative and postoperative periods, irrespective of the magnitude of tumor response”, because “…this will increase the probability of achieving pCR, which is a proven factor for a good prognosis…”. We hypothesize that the above recommendations and trends in routine clinical practice are the consequences of misunderstanding regarding the concept of pCR, which has led to a shift in its importance from a prognostic factor to a desired treatment outcome. The origin of this misunderstanding could be a strong subconscious incentive to achieve pCR, as patients who achieved pCR after NAST had a better long-term outcome compared with those who did not. In this paper, we attempt to prove our hypothesis. We performed a comprehensive analysis of the therapeutic effects of NAST and adjuvant systemic therapy (AST) in EBC to determine whether pCR, as a phenomenon that can only be achieved at NAST, improves prognosis per se. We used published papers as a source of data, which had a decisive influence on the formation of the modern attitude towards EBC therapy. We were unable to find any evidence supporting the use of pCR as a desired therapeutic goal because NAST (reinforced by pCR) was never demonstrated to be superior to AST in any context.

## Introduction

A positive correlation between pathohistologically confirmed complete elimination of tumor tissue from the breast and axillary lymph nodes by preoperative systemic therapy (pathological complete response – pCR) and a favorable disease outcome was first observed at the turn of the century in early clinical trials on the therapeutic effect of neoadjuvant systemic therapy (NAST) (1–3). Several meta-analyses confirmed these observations (4, 5). Based on these findings, the idea of using pCR in clinical studies as a surrogate endpoint for long-term outcomes (LTOs) was born (6). This was motivated by the desire to shorten the length of time necessary to obtain information on the therapeutic effects of new drugs. Whether pCR is achieved can be determined within a few months after starting treatment, but gathering information on LTOs takes several years. In clinical trials, LTOs are best represented by one of the following parameters: event-free survival (EFS), disease-free survival (DFS), distant-disease-free survival (DDFS), and overall survival (OS), depending on the predefined primary research goals.

It is undeniable that pCR is valuable in clinical studies when comparing the therapeutic effects of different doses and regimes of systemic therapy (the *in vivo* chemosensitivity test), testing the effect of new medications, determining the effects of systemic therapy in different biological subtypes of breast cancer, evaluating the need for more aggressive adjuvant treatment after NAST, etc (7–13). Numerous randomized clinical trials (RCT), (around 40% of RCTs, based on the U.S. National Library of Medicine data) of early breast cancer (EBC) treatment have recently evaluated the use NAST as a primary therapeutic intervention and pCR as a primary study endpoint (14). This especially relates to the breast cancer subtypes which achieve the highest percentage of pCR (HER2-positive breast cancer and triple-negative breast cancer [TNBC]) (15) and simultaneously show the highest level of positive correlation between achieving pCR and favorable LTOs (16, 17). As a consequence of these developments, pertuzumab would became the first drug to be registered for use in early breast cancer neoadjuvant settings based solely on pCR data from the Tryphaena and NeoSphere trials (7, 18).

In recent years we have witnessed a tendency to treat pCR as a treatment goal in routine clinical practice, not only as one of pieces of information gleaned from clinical trials. These tendencies in routine clinical practice are the result of recommendations given in treatment guidelines which is vividly illustrated by the following statements found in the ESMO’s EBC treatment guidelines: “…If ChT (chemotherapy) is used (preoperatively), it is recommended to deliver all planned treatment without unnecessary breaks, i.e. without dividing it into preoperative and postoperative periods, irrespective of the magnitude of tumor response. This will increase the probability of achieving pCR, which is a proven factor for a good prognosis…” (19).

Novel and more extensive meta-analyses reaffirmed a statistically significant correlation between achieving pCR and favorable LTOs especially in HER2-positive cancers and TNBC (20, 21). Despite this, evidence for the validity of pCR as a surrogate endpoint at a trial-level remained conspicuously absent (22–26). Hence, some authors expressed doubts about the value of pCR to be used as a study endpoint as part of accelerated drug registration efforts (22–24).

In this text, we focus on above-mentioned tendencies and recommendations to treat pCR as a goal of breast cancer therapy in routine clinical practice, the justifiability of tailoring treatment strategies based on these tendencies and recommendations, and potential negative clinical implication for patient interest of such an approach. Our working hypothesis is that pCR is useful prognostic information, not a source of good prognosis.

We used English language meta-analyses as well as the individual studies they analyzed that have been published in the past 30 years as a source of data on pCR and the therapeutic effects of NAST and AST. Reviews were also included if they reported on data pertinent to the subject we are analyzing. Since numerous publications deal with the subject matter of interest, we cited only the largest and most significant studies that have had a crucial effect on the formation of modern attitudes towards early breast cancer therapy as well as studies we deemed successfully illustrate and elaborate on our thinking. All the studies were identified by a PubMed search.

## Conditions for pCR to satisfy in order to be considered a “factor for a good prognosis”

In a clinical situation, the issue of the difference between the terms “a good prognostic factor” and “a factor for a good prognosis” comes down to function. A prognostic factor is a parameter, which, with a satisfactory level of reliability, serves to predict the ultimate outcome of a disease without actually influencing said outcome. A good prognostic factor is an information parameter, which, with a satisfactory level of reliability predicts the outcome - without actually influencing the final outcome, while a factor of (for) a good prognosis represents a clinical state or clinical activity that actively influences the improvement of the prognosis. For instance, the stage of the disease at the moment when treatment is started is just information on the expected outcome, while the treatment that is carried out represents a factor that will improve the prognosis (as compared to, for example, not carrying out treatment).

Based on the complete formulation of the recommendation from the ESMO guidelines, it is impossible to eschew the impression that pCR is to some extent perceived as a parameter that actively influences the prognosis, which is clear from the above-stated quote. This, however, can be considered a valid premise only if certain conditions are met. In the following text we list the conditions that would need to be fulfilled for pCR to be considered a factor which directly influences prognosis:

a) If pCR were an independant factor influencing favorable outcomes of breast cancer treatment, this influence would be observed with comparable intensity in all breast cancer subtypes. This, however, is not the case. In a pooled analysis of seven German trials, performed on 4,193 patients, in the luminal A and luminal B/HER2-positive types, those who achieved pCR had practically the same OS as those who did not. On the other hand, in patients with luminal B/HER2-negative, non-luminal/HER2-positive, and TNBC, LTOs were significantly better in patients who had achieved pCR (10). (**Table 1**).

**Table 1:** Differences in DFSs between patients who have attained pCR compared to those who did not, by cancer subtype [Kaplan-Meier curves and Log rank tests were utilized) (the table was extrapolated from the data published by Minckwitz et al. (11)].

Cancer subtype	N (total)	N with pCR	N without pCR	Percent of patients achieving pCR (%)	p
Luminal A	1,637	105	1,532	6.4	0.39
Luminal B Her2 negative	357	40	317	11.2	**0.005**
Luminal B Her2 positive	751	126	625	16.8	0.45
Her2 enriched	537	164	373	30.5	**< 0.001**
TNBC	911	282	629	31	**< 0.001**

Statistically significant values are bolded.

In the CTNeoBC meta-analysis, the positive association between pCR and a favorable LTO was stronger in high-grade tumors, as compared to low-grade tumors, while the most favorable LTOs were observed in patients with HER2-positive tumors treated with trastuzumab and in those with TNBC (23). The situation is similar in different molecular subtypes of TNBC, wherein, for instance, the BL1 molecular subtype achieves a significantly higher percentage of pCR, as compared to the LAR and BL2 subtypes, with the LAR subtype having the best prognosis, irrespective of the relatively low percentage of pCR (27, 28). In a meta-analysis of the relationship between pCR and OS in different molecular breast cancer subtypes on 13,939 patients Haque et al. found a statistically significant difference in achieving pCR amongst the different subtypes (luminal A – 0.3%; luminal B – 8.3%; HER2-positive – 38.7%; TNBC – 23.2%) (29). Interestingly, the prognosis differed even among the subgroup of patients who had had achieved pCR. OS was statistically significantly longer in pCR patients with HER2-positive cancers compared to those with TNBCs (p = 0.016).

b) pCR should be a phenomenon which is, in terms of the surrogate endpoint, independent in relation to other quantitative levels of therapeutic response. However, the correlation between pCR and LTO is a part of the continuous correlation between tumor responses to systemic therapy and LTO, which is why viewing the pCR as a separate prognostic parameter is a deficient approach. In the EBCTCG meta-analysis of NAST, as well as in the meta-analysis of the influence of residual cancer burden on prognosis carried out by Yau et al, LTO consistently and statistically significantly positively correlated with other levels of responses (usually considered to be suboptimal) to systemic therapy, like partial response, stable disease, etc (30, 31).
c) As such, pCR should represent the single most specific characteristic of the tumor, which we could most closely describe as the “propensity of the tumor to respond to systemic therapy” (PTR – propensity of tumor response). PTR should be distinguished from chemosensitivity, because the heterogeneous impact of this particular characteristic on prognosis in different types of breast cancer speaks in favor of the fact that chemosensitivity is not the only aspect of this characteristic. Rather, this characteristic represents a synergy of a number of combined (often unknown) factors of prognosis, whose volume of influence is unevenly distributed in different types of breast cancer (24). (**Table 2**).

**Table 2:** The association of residual cancer burden (RCB*) categories with EFS [extrapolated from Yau et al. (31)].

Residual cancer burden category	EFS (%)	95% CI
RCB-0	91	90 – 93
RCB-1	86	84 – 89
RCB-2	74	72 – 76
RCB-3	58	54 – 62

*The residual cancer burden (RCB) is a continuous variable that represent the percentage of tumor tissue remaining after neoadjuvant therapy. Empirically derived cut-off points to define four RCB classes, from RCB-0 to RCB-3, representing increasing residual disease. PCR would then correspond to RCB-0 (31).

A substantial percent of the patients who have had experienced pCR, should have a favorable LTO. However, an appreciable number of patients with a pCR experience recurrence of the disease or die during follow-up. Also, a certain percentage have a favorable outcome without having achieved pCR. Hence, focusing exclusively on achieving pCR during breast cancer treatment can be potentially harmful.

d) If pCR were an independant factor influencing a favorable outcome, as a clinical phenomenon which can be achieved only within NAST, any given regimen of NAST should achieve better therapeutic effects as compared to when the same drug/drugs is/are used as AST in similar cohorts and for tumors with the comparable clinical and molecular characteristics at baseline. This difference should be proportionately the more pronounced in tumors and therapeutic regimens that achieve the highest percentages of pCR (e.g. HER2-positive receiving targeted therapy and TNBC).

In the following three chapters, we discuss this last item (d) in detail

## NAST vs AST in early clinical trials

One of the recurring themes in the early clinical trials of NAST, carried out in the 1980s, 1990s, and the first decade of the 21^st^ century, was the lack of patient selection based on biological tumor profiling into subtypes. Also, hormone and anti-HER2 therapy received relatively little attention. Study groups were balanced according to baseline patient clinical characteristics and disease stage, receiving the same regimen systemic therapy before or after surgery. Four relevant meta-analyses analyzed the results of a total of 16 studies, some of which are included in more than one meta-analysis due to the time lag between them and a tendency to include all the relevant data in the meta-analyses (1–5, 30, 32–55). (**Table 3**).

**Table 3:** Characteristics of the studies utilized in meta-analyses.

		Meta-analysis							
Study	Reference	Mauri	Mieog	EBCTCG	Chen	Total number of patients	pCR (%)	Duration of follow-up (months)	Difference in LTOs
S6	Scholl 1991 (34)	√	√	√	√	200/190	/	105	NS
	Scholl 1994 (35)	√			√				
	Broet 1999 (36)	√	√		√				
NSABP	Fisher 1998 (37)	√			√	763/760	13%*	114	NS
	Wolmark 2001 (2)	√	√	√	√				
	Rastogi 2008 (38)	√			√				
EORTC	Van der Hage 2001 (1)	√	√	√	√	350/348	4%	120	NS
	Van Nes 2009 (39)				√				
ECTO	Gianni 2005 (32)		√			451/451	20%	76	NS
	Gianni 2009 (40)			√	√				
IBBGS	Mauriac 1991 (41)				√	134/104	/	124	NS
	Mauriac 1999 (42)	√	√	√	√				
Royal Marsden	Powles 1995 (43)			√		149/144	10%	112	NS
	Makris 1998 (44)	√			√				
	Cleator 2005 (3)		√		√				
ABSCG	Jakesz 2001 (53)		√			203/195	5.9%	108	NS
	Taucher 2008 (45)			√	√				p=0.024^a^
St. Petersburg	Semiglazov 1994 (46)	√	√		√	137/134	29%	53	NS
Edinburgh	Forouhi 1995 (47)		√		√	40/39	/	/	NS
Japan	Enomoto 1998 (48)		√		√	20/25	/	24	NS
Lithuania	Ostapenko 1998 (49)		√		√	50/50	/	42	NS
London	Gazet 2001 (50)	√	√	√	√	100/110	/	60	NS
USA	Danforth 2003 (51)	√	√	√	√	26/27	20%	108	NS
	Deo 2003 (52)				√	50/51	/	25	NS
	Liu 2010 (54)				√	264/258	/	67.4	NS
Vancouver	Ragaz 1997 (55)					42/39		/	NS

a DFS superior in the AST group.

The primary goal of all of the 16 aforementioned trials, as well as the four meta-analyses, was to compare the therapeutic effects (DFS and OS) of preoperative and postoperative application of systemic therapy (NAST vs AST). The secondary goals of the trials and meta-analyses included the following: assessment of the magnitude of the therapeutic response to NAST by determining the clinical and pathohistological response, comparison of the scope of surgical procedures between the NAST and the AST groups (breast conserving vs mastectomy), determining the frequency of local recurrence in the two groups, and analysis of the toxicity of the applied regimens.

The authors of all four meta-analyses, Mauri et al. (9 processed trials with a total of 3,946 patients), Mieog et al. (14 processed trials with a total of 5,500 patients), EBCTCG (10 processed trials with 4,756 patients) and Chen et al. (16 processed trials with 5,593 patients), emphasize the uniformity of the analyzed groups, with respect to baseline clinical characteristics of both patients and tumors, as well as the preoperative and postoperative systemic therapy in each individual trial (4, 5, 30, 33). None of the meta-analyses were able to demonstrate a significant difference in the total DFSs or OSs (at either the trial or patient level) between those who had received chemotherapy before surgery compared to those who had received the same chemotherapeutic after surgery (4, 5, 30, 33). Put simply, NAST and AST yield identical OSs and DFSs. Looking at the NAST subgroup alone, patients who achieved pCR had a significantly longer OS and DFS, as compared to the patients who did not achieve pCR (5, 33). The percentage of patients who had achieved pCR ranges from 4% to 20% (in a study by Semiglazov et al. the percentage those achieving pCR after NAST is 29%; however, in this study, neoadjuvant therapy included preoperative radiation therapy) (1, 32, 46).

The EBCTCG meta-analysis compares LTOs between each of the three NAST subgroups, obtained by stratification based on the magnitude of the clinical response (546 complete clinical responders, 803 partial clinical responders and 598 non-responders) and the entire AST group (30). There is a statistically significant negative correlation between the magnitude of the clinical response and the probability of disease recurrence and lethal outcome, i.e. a better clinical response to NAST decreases the probability of recurrence and lethal outcome, and vice versa. Complete clinical responders have a statistically significantly better OS, as compared to the entire AST group (p = 0.001); partial responders have a similar OS that of the entire AST group (p = 0.96); while non-responders have a statistically significantly poorer OS, as compared to the entire AST group (p = 0.00001).

The following could be discerned by reviewing the results of the four aforementioned meta-analyses:

1. The NAST and AST groups were uniform regarding baseline patient and tumor characteristics (age, hormone receptor status, tumor size and grade, lymph node status, etc).
2. Every individual trial analyzed only a single therapeutic regimen with one subgroup receiving it preoperatively and the other postoperatively.
3. Each individual trial reported on all magnitudes of clinical response during NAST, i.e. complete response, partial response, stable disease and progressive disease.
4. The existence of a certain percentage of pCR (which is always above zero) in each trial reporting on that information.
5. The existence of a statistically significant positive correlation between clinical response to systemic therapy and LTOs was demonstrated, independently of whether the response to therapy is assessed with clinical response or with pCR.
6. The lack of a statistically significant difference in LTOs between the overall NAST and AST groups was also demonstrated.

These observations lead us to conclude the following:

1. Patients with early breast cancer and the same baseline characteristics have similar LTOs irrespective of whether systemic therapy is applied prior to (NAST) or after (AST) the surgical removal of cancerous breast tissue and regional lymph nodes.
2. The response to NAST can be evaluated using clinical diagnostics and is based on cancerous breast tissue shrinkage/disappearance, while this remains impossible in AST patients because the tumor has, by definition, already been removed before the start of adjuvant systemic therapy.
3. There is a significant positive correlation between therapeutic response to NAST and treatment outcome.
4. The only difference between NAST and AST is the magnitude of data on the clinical and pathohistologic therapeutic response to NAST, available to oncologists.
5. It is only logical that the AST group would have had the same distribution of clinical and pathohistologic responses as the NAST group, if in AST group had systemic therapy been given prior to surgery. The same holds true in the other direction – the NAST group would have probably had the same LTO had they received the chemotherapy after (instead of prior to) surgical removal of cancerous tissues.
6. The percent of patients achieving pCR, as well as the magnitude of the clinical response to preoperative systemic therapy, represents information, but in no way represents the source of a good prognosis.

## NAST VS AST in HER2-positive breast cancer

Thirty to seventy-five percent of patients with HER2 positive breast cancer achieved pCR following neoadjuvant chemotherapy combined with HER2-targeted therapy - a significantly higher percent compared to neoadjuvant chemotherapy of breast cancer overall (all breast cancer subtypes combined) which attain a pCR of 20% at its most successful (4, 5, 32, 33, 56, 57). The percent of achieved pCR in HER2-positive breast cancers is positively correlated with more favorable LTOs (16, 20, 58). These facts considered, one might expect the positive effect of pCR on a favorable prognosis, if it indeed exists, to be most evident in the HER2-positive subpopulation. In other words, neoadjuvant treatment of HER2-positive breast cancer should lead to a more prominent improvement in prognosis compared to adjuvant chemotherapy with the same drug than neoadjuvant treatment of other breast cancer subtype, if pCR were a factor independently influencing a favorable prognosis. Some insight into this question can currently be gleaned from available randomized clinical trials and meta-analyses focused on HER2-positive breast cancer management. However, clinical trial dynamics and various shareholder interests, as well as a previously demonstrated noninferiority of NAST compared with AST in subtype-nonspecific trials, probably led to a paucity of clinical trials comparing anti-HER2 therapies in a neoadjuvant versus an adjuvant setting (4, 5). Most studies concerning anti-HER2 monoclonal antibodies either compared chemotherapy/trastuzumab combination therapy to chemotherapy alone or chemotherapy/dual anti-HER2 therapy to chemotherapy/trastuzumab. All these studies were conducted in either an adjuvant or a neoadjuvant setting (7, 59–70). However, since all studies had a chemotherapy/trastuzumab arm, it is possible to make observations about the therapeutic success of this therapeutic regimen in a neoadjuvant compared with an adjuvant setting. Neoadjuvant therapy versus adjuvant therapy data from the chemotherapy/trastuzumab arms of these studies are contrasted in **Table 4**.

**Table 4:** NAST vs AST in HER2-positive breast cancer.

	Trial	Number of patients (cht/T branch)	Cumulative follow-up (patient-years)	Mean follow-up (years)	Patients with N0	Percent of patients with N0 (%)	Number of patients not experiencing an event	EFS (%)	pCR (%)
Trials of AST	Aphinity	2,405	14,430	6	902	37.5	2,124	88.3	/
	Fin Her	115	12,305	10.7	18	16	79	68.7	/
	NSABP B31	1,058	9,945.2	9.4	0	0	837	79.1	/
	NCCTE (Romona)	2,273	29,094.4	12.8	295	13	1,787	78.6	/
	HERA	3,402	37,422	11	1,087	32	2,497	73.4	/
	PACS04	260	2,496	9.6	0	0	186	71.5	/
	BCIRG006	1,074	11,277	10.5	311	29	877	81.7	/
	Sum	10,587	105,895.3	10	2,613	24.7	8,387	79.2	
Trials of NAST	NOAH	117	631.8	5.4	16	14	68	58	38
	NeoSphere	107	535	5	32	30	87	81	29
	NSABP B41	177	885	5	87	49	149	84.3	52.5
	NeoALTTO	149	561.73	3.77	/	84.6^a^	113	76	29.5
	TAKADA	829	2,487	3	269	32.5	721	87	51
	TECHNO	217	651	3	77	35.5	169	78	39
	HANNAH	596	2,086	3.5	134	22.5	447	75	34
	Sum	2,192	7,837.53	3.6	615^b^	30	1754	80	

a N0 + N1.

b The number of patients with N0 stage cancer was not reported on in the NeoALTTO trail.

The high percentage of pCRs achieved by the chemotherapy/trastuzumab protocol in the neoadjuvant setting did not translate to a more favorable LTOs than when the same protocol was applied in adjuvant settings. The mean number of patients that had not experienced a breast cancer-related event during follow-up in all AST groups was calculated to be 79.2%, as compared to 80% in the NAST groups. However, one would do well to notice that the median follow-up in the neoadjuvant group was 3.6 years, and almost three times as long in the adjuvant group (10 years). Given that the percent of patients that have not experienced a breast cancer-related event decreases with the passage of time, it would have likely been much lower than the one observed had follow-up been prolonged. As an observational measure of the initial stage of disease, N0 stage was present in 24,7% of patients in the adjuvant therapeutic trials, compared to 30% in neoadjuvant trials, leading us to conclude that the cancer stages in the neoadjuvant group were, at least, no more advanced than in the adjuvant group.

The biggest meta-analysis of the therapeutic effects of NAST in HER2-positive breast cancer (irrespective of the composition of the therapeutic protocol) conducted thus far (78 studies and 25,150 patients were included) computed an overall pCR frequency of 40.9% (20). This meta-analysis provides a stratified presentation of the EFS percentages, in relation to the follow-up period within the individual studies. For instance, the five-year EFS was estimated on the basis of data from 19 studies which reported on five-year EFS, in 6,675 patients. The average pCR in these 19 studies was 38.3% (2,559 patients with pCR and 4,116 patients with residual disease), while the overall five-year EFS was 74% (number of events: 1,736/6,675 = 26%). In EBCTGC’s meta-analysis of 13,864 patients with a median follow-up of 10.7 years over 6 trials of adjuvant trastuzumab and a single trial of neoadjuvant trastuzumab, the overall percentage of people not experiencing a breast cancer-related event by the end of follow-up was 76.5% (61, 64, 65, 71). In this comparison of two meta-analyses one should also bear in mind the great difference in the follow-up period, which additionally emphasizes the non-inferiority of AST as compared to NAST, in a situation when the EFS percentages have similar values.

All the aforementioned data point to the fact that AST is at least non-inferior compared to NAST when it comes to therapeutic efficacy in HER2-positive breast cancer patients. This holds true despite an achieved impressive 40% pCR in the NAST group.

## NAST vs AST in patients with triple negative breast cancers

TNBCs are a heterogeneous group of breast cancers with several subtypes differentiated based on both genetic, molecular and immunologic characteristics, and clinical behavior when exposed to systemic therapy as well as prognosis alike. A common characteristic of TNBCs is a low level of hormonal and HER2 receptor expression on the surface of tumor cells making chemotherapy the cornerstone of treatment (27, 28, 72). The poor overall prognosis coupled with a good response to systemic therapy spurred researchers’ efforts to increase its efficacy, enrich (Capecitabine, Platinum salts, etc) and intensify it and to add on immunotherapies (i.e. pembrolizumab), PARP inhibitors (Olaparib) and other drugs.

Said good responsiveness to chemotherapeuticals (anthracyclines, taxanes, and cyclophosphamide) was confirmed in a neoadjuvant setting as well with a mean of 35% of patients reaching pCR (72). As is the case in HER-enriched breast cancers, a significant positive correlation between pCR and survival was unambiguously demonstrated in patients with TNBC. A meta-analysis by Huang et al. found pCRs to range from 16.7% to 67% (17). Total five-year EFS in this study was 86% after NAST if the patients had achieved pCR and 50% if they had failed to do so. Five-year OS was 92% and 58% in these two groups, respectively. However, this vast difference in LTOs between the pCR and non-pCR subgroups cannot be taken to mean that NAST is superior to AST. Two of the biggest studies making comparisons between the therapeutic performances of AST and NAST are two retrospective analyses of the data of the US National Database, one conducted from 2010 to 2011, and the other spanning four years (from 2010 to 2013) (73, 74). Despite the fact that pCR in the newer four-year study was found to be an impressive 47.4%, in both studies survival was statistically significantly more common in the AST group compared to the NAST group (87.5% vs 75.8% in a sample of 15,483 in the older and 76.8% vs 73.4% in a sample of 19,151 patients in the newer study) (74). Cheng et al. found that the patients in the NAST group had more advanced tumors compared to the AST group (35%: 6% stage III cancers, respectively). They used stabilized inverse proportion weighing to adjust for this confounding factor and calculated that the adjusted OS was still significantly longer in the AST group (85.4% compared to 81.9%) (73).

The only meta-analysis comparing the effects of AST and NAST in TNBC was conducted on a sample of 36,480 patients and showed that those that had been treated with AST had an improved overall survival compared to those who had received NAST (HR = 1.59; 95% CI = 1.25–2.02; p = 0.0001) even though the average frequency of pCR was 35% (75). Patients achieving pCR had a significantly prolonged overall survival when compared to AST in all the studies included in the meta-analysis (HR = 0.53; 95% CI = 0.29–0.98; p = 0.04). Patients who had failed to attain pCR had a significantly worse overall survival when compared to AST (HR = 1.18; 95% CI = 1.09–1.28; p < 0.0001).

## Lost in translation

- The information about a positive correlation between pCR and a favorable LTO comes as a result of a counter-intuitive (one might say unnatural) division of the entire NAST group into the pCR and non-pCR subgroups. A better prognosis in one subgroup, necessitates a worse one in the other (30, 75).
- If the propensity of a tumor to achieve pCR is an indication for NAST in and of itself, then the propensity of a tumor for not achieving pCR should represent a contraindication for NAST, because the non-pCR subgroup has a significantly worse LTO compared to the entire AST group.
- Large tumors (the locally advanced stage) are less likely to attain pCR compared to smaller tumors (29, 76). This brings us to a paradox – the original indication for NAST and the one that ensured its induction into clinical practice, i.e. the improvement in feasibility of surgical treatment of locally advanced disease and higher percent of breast conserving surgeries (BCSs), now seemingly becomes a relative contraindication for NAST (30).
- The PTR in all patients receiving NAST and all quantitative levels of therapeutic response can be determined by modern imaging and laboratory diagnostics, novel on-treatment biomarkers of the chemotherapy response like AAGAB, which may offer a more adequate surrogate endpoint compared to achieving pCR (3, 77–80). Marczyk et al. have recently developed the treatment efficacy score (TES) based on the quantitation of the residual cancer burden (81). They demonstrated that TES is superior to pCR as a surrogate marker for favorable LTOs since it, amongst other things, captures all the nuanced levels of therapeutic response, not only the pCR.
- The PTR is potentially manifest even in the early weeks of NAST application, i.e. the therapeutic response to NAST can be accurately assessed via MRI and/or ultrasound in the early phases of therapy, before pCR is actually attained (77, 78).
- An achieved pCR implies the loss of useful information that could be obtained by analyzing the residual tumor tissue. Also, the residual tumor is the best possible marker for the extent of surgical resection.
- The misconceptions in relation to the concept of pCR stem from a psychological trap, which has led to a shift of its meaning from a prognostic factor to a desired treatment outcome. Namely, the fact that patients who have achieved pCR following NAST have better LTOs compared to those who did not is a powerful subconscious incentive to make great efforts and achieve pCR. Furthermore, there is apparently a subconscious psychological tendency to make a conceptual confusion between two senses of the term “factor”: pCR as a good prognostic factor and, mistakenly, an efficient causal factor. Indirectly, this becomes a tendency towards an as extensive and as prolonged a NAST as possible because pCR can only be attained within the purview of NAST. In this way, the psychological influences and subconscious dynamics have led to committing the logical fallacies of straw-man argument and ignoratio elenchi.

## Discussion

The question of whether pCR is a prognostic factor only or even a desired outcome per se is not just a matter of semantics but has implications larger than purely academic ones. It directly influences the treatment quality for a large number of breast cancer patients worldwide. If we analyze the structure of ESMO’s recommendation that wholesome systemic therapy before surgery should be reverently applied “irrespective of the magnitude of tumor response” we must conclude that strictly adhering to this recommendation will be deleterious to a certain percent of patients whose tumors are poorly responsive to systemic therapy, since they will unduly waste precious time by delaying life-saving surgery. If non-responder data from early NAST trials is anything to go by, the percent of patients that could be harmed by such reasoning amounts to approximately 30% (30). This prevents any kind of utilitarian justification for the ESMO recommendation, and it is very difficult to see what other type of moral justification might be attempted.

The broad modern-day NAST indications are a consequence of the predicted good therapeutic responsiveness of certain breast cancer subtypes and therapy enrichment with new biologicals, the combination of which have significantly reduced the percent of non-responders. However, NAST indications are still dominantly reliant on initial disease staging, i.e. the main indication for NAST is the presence of an inflammatory or locally advanced breast cancer disregarding the biological subtype of the cancer (19, 82). It has been demonstrated that tumors that are larger at diagnosis have a subpar response in the pCR context, compared to smaller tumors regardless of their subtype (29, 76). If we want to remain consistent with the original indications for NAST - tumor reduction for the purpose of more optimal surgical treatment, insisting on achieving pCR is counterproductive.

We posit that delaying surgery is deleterious in patients in whom NAST might be ineffective. This subject has only been hypothesized about by some authors and it has never been, to our knowledge, specifically covered by a stand-alone publication up to this point (3, 5, 33). We base our claim on the basic and time-honored axiom of oncology that advanced disease portends a poorer prognosis, a fact that the inviolable TNM staging system is also based on. Other than the influence of the intrinsic biologic aggressiveness, in some advanced malignant disease is a consequence of time lost before a diagnosis was established and the adequate treatment initiated. Another fact that lends credence to our claim is the favorable influence of breast cancer screening programs on survival since these benefits are entirely a consequence of early diagnosis and treatment (83).

Our comparative observational analysis of the therapeutic effects of NAST and AST did not identify any data that could be considered proof of the superiority of NAST, even in breast cancer subtypes with a high pCR rate. We believe this to be cause enough to reevaluate pCR as a parameter that guides therapeutic decisions in routine clinical practice. Flexible targeted and individualized therapy that combines systemic and surgical treatment (with the awareness that the effects of systemic therapy are equally effective in preoperatively, postoperatively or partially preoperatively and partially postoperatively setting) could conceivably offer a better chance of survival to all patient subgroups, especially those that do not respond to systemic therapy.

PCR is a prognostic factor that indicates a good prognosis but it must not be used as a therapeutic goal in the context of all and any breast cancer therapeutic modalities. The utilization of pCR as a therapeutic goal in routine clinical practice does not offer any benefit to the subpopulation of patients that manage to attain it, but significantly harms those in whom pCR remains elusive. This is a consequence of delaying necessary and unambiguously useful surgical management. Using pCR as a source of prognostic information in clinical trials may make sense in some aspects but it does not justify its misuse as a therapeutic goal in routine clinical practice.

Conclusion: The observed use of pCR as a therapeutic goal in the treatment of early breast cancer is not justified and may be harmful to a significant percentage (pool) of patients with modest therapeutic responses to systemic therapy. Therapeutic recommendations based on achieving pCR as a therapeutic goal should be carefully reevaluated.

## Data availability statement

The original contributions presented in the study are included in the article/supplementary material. Further inquiries can be directed to the corresponding author.

## Author contributions

NI contributed to the study conception and design, analysis, interpretation of results and drafted the manuscript. DB, BL, NC, SP, MG, OZ, KZ and DZ contributed to data collection, analysis and interpretation of results. MB contributed to ethical and psychological considerations. All authors contributed to the article and approved the submitted version.
